# Efficacy and Safety of Subcutaneous Rocatinlimab in Adults With Moderate‐to‐Severe Atopic Dermatitis: A Systematic Review and Meta‐Analysis of Randomised Placebo‐Controlled Trials

**DOI:** 10.1155/drp/2160445

**Published:** 2026-09-20

**Authors:** Hong Jing Wang, Leong Seng Wang, Jordan Wei Lun Tan, Ee Syuen Wang, Yan Ye Lee

**Affiliations:** ^1^ Department of Medicine, Flinders Medical Centre, Bedford Park, Adelaide, South Australia, Australia, flinders.sa.gov.au; ^2^ School of Pharmacy, Monash University Malaysia, Bandar Sunway, Selangor, Malaysia, monash.edu.my; ^3^ Mackay Base Hospital, Mackay, Queensland, Australia, health.qld.gov.au

**Keywords:** anti-OX40 therapy, atopic dermatitis, biological therapy, eczema, rocatinlimab

## Abstract

**Background:**

Atopic dermatitis (AD) is a relapsing, chronic inflammatory skin disease. Rocatinlimab, an investigational anti‐OX40 monoclonal antibody, has been evaluated for moderate‐to‐severe AD. This systematic review and meta‐analysis aimed to evaluate the short‐term efficacy and trial‐reported safety of subcutaneous rocatinlimab in patients with moderate‐to‐severe AD.

**Methods:**

We searched PubMed, Ovid MEDLINE, Embase, Cochrane Central Register of Controlled Trials (CENTRAL) and ClinicalTrials.gov to identify randomised controlled trials (RCTs) comparing rocatinlimab with placebo in patients with moderate‐to‐severe AD. Only summary‐level data from published reports were analysed. Outcomes of interest included treatment efficacy and safety. A random‐effects model with Hartung–Knapp–Sidik–Jonkman adjustment was used for the primary pooled analyses. Exploratory subgroup analyses were conducted according to both dose and dosing frequency, with Wald‐type confidence intervals used for subgroup‐specific pooled estimates involving only two studies.

**Results:**

A total of 1767 patients from 3 RCTs were included. A total of 1305 patients (73.85%) received subcutaneous rocatinlimab. Rocatinlimab was associated with a significantly greater achievement of the pooled estimate for EASI‐75 at Week 16 (OR 3.58, 95% CI 1.53–8.40; *p* = 0.02). The pooled estimate for the vIGA‐AD score of 0 or 1 at Week 16 was directionally favourable but imprecise and did not reach statistical significance (OR 4.06, 95% CI 0.56–29.47; *p* = 0.09) No statistically significant differences were observed in serious adverse events (OR 1.60, 95% CI 0.41–6.26; *p* = 0.28) or treatment discontinuation due to adverse events (OR 1.28, 95% CI 0.14–11.60; *p* = 0.68), although confidence intervals were wide.

**Conclusion:**

In adults with moderate‐to‐severe AD, subcutaneous rocatinlimab demonstrated potential short‐term treatment efficacy compared with placebo. However, the available evidence does not establish durable remission, prevention of recurrence, long‐term disease modification or a favourable long‐term benefit–risk profile. Given the subsequent discontinuation of the rocatinlimab clinical development programme following safety review, these findings should be interpreted cautiously as a synthesis of short‐term trial evidence rather than support for future clinical use.

## 1. Introduction

Atopic dermatitis (AD) is a chronic, relapsing inflammatory dermatological disease affecting at least 230 million people globally [1, 2]. AD is a combination of immune dysregulation and skin barrier dysfunction, presenting with intense pruritic and erythematous lesions, xerosis and lichenification [1]. The itch–scratch cycle of AD amplifies the inflammatory pathways and aggravates skin barrier dysfunction. Furthermore, chronic itching and visible lesions may cause social isolation and poor sleep quality that lead to emotional distress, a reduction in quality of life and a burden of disability among skin diseases [3].

The systemic treatment landscape for moderate‐to‐severe AD has expanded considerably in recent years. Current options include biologic monoclonal antibodies, such as dupilumab, tralokinumab and lebrikizumab, and oral small‐molecule targeted therapies, such as Janus kinase (JAK) inhibitors, including upadacitinib and abrocitinib [4, 5]. Biologic monoclonal antibodies generally target extracellular cytokines or cytokine receptors involved in Type 2 inflammation, whereas JAK inhibitors act intracellularly to modulate cytokine signalling pathways [4, 5]. Despite these advances, not all patients achieve adequate disease control, some experience adverse effects or contraindications and questions remain regarding optimal treatment sequencing, long‐term disease control and safety across different systemic treatment options [4, 5].

On the other hand, the OX40 ligand pathway has been investigated as a potential therapeutic target in T‐cell costimulation and inflammatory persistence [6]. OX40 is expressed on activated effector and memory T cells, as well as on a subset of regulatory T cells that appears altered in AD [7]. Rocatinlimab is a humanised monoclonal antibody directed against the OX40 receptor that depletes this OX40‐expressing T‐cell population [6, 8]. However, this mechanistic rationale should be interpreted cautiously. Clinical responses have only been shown to persist for a limited period after treatment stops; therefore, long‐term disease control has not been established [8]. In addition, because OX40 is found on both effector T cells and regulatory T cells, depleting these cells nonselectively could plausibly affect the body’s normal immune surveillance, which is relevant to the malignancy concerns that later contributed to discontinuation of the rocatinlimab programme [7, 9].

Recent randomised controlled trials (RCTs), including a Phase 2b study and the ROCKET‐IGNITE and ROCKET‐HORIZON Phase 3 trials, have reported clinical efficacy and safety outcomes for rocatinlimab in adults with moderate‐to‐severe AD [8, 10]. These trials provide important short‐term evidence, but the available clinical evidence remains limited to a small number of placebo‐controlled studies with differences in trial phase, dosing regimen, treatment duration and outcome reporting [8, 10]. A systematic review and meta‐analysis is therefore useful to synthesise the totality of randomised evidence, generate pooled estimates for key efficacy and safety outcomes, assess the consistency of findings across available trials and evaluate the certainty and limitations of the current evidence base [11, 12].

Since the completion and publication of the pivotal rocatinlimab trials, the development status of rocatinlimab has changed substantially. In January 2026, Kyowa Kirin announced termination of its development and commercialisation collaboration with Amgen, with Kyowa Kirin regaining control of the global rocatinlimab programme [13]. Subsequently, in March 2026, Kyowa Kirin announced discontinuation of all ongoing rocatinlimab clinical trials following a planned safety review, stating that the overall benefit–risk profile was no longer considered favourable in the studied populations [9]. These developments are highly relevant to the interpretation of the available trial data, particularly because short‐term randomised trials may not fully capture rare, delayed or long‐latency safety outcomes such as malignancy [9, 14].

Accordingly, this systematic review and meta‐analysis should not be interpreted as supporting current clinical adoption of rocatinlimab. Rather, it aims to synthesise the available randomised placebo‐controlled evidence on short‐term efficacy and trial‐reported safety outcomes of subcutaneous rocatinlimab in adults with moderate‐to‐severe AD and to interpret these findings in light of the limited long‐term evidence base and subsequent safety‐related discontinuation of the clinical development programme.

## 2. Methods

This systematic review was conducted and reported in accordance with the Preferred Reporting Items for Systematic Reviews and Meta‐analyses (PRISMA) guidelines (PRISMA checklist is available in Supporting Information S1) [11]. The review protocol was not prospectively registered in PROSPERO or another public systematic‐review registry.

### 2.1. Search Strategy

A comprehensive database search was conducted across PubMed, Ovid MEDLINE, Embase, Cochrane Central Register of Controlled Trials (CENTRAL) and ClinicalTrials.gov from inception up to 31 January 2026. The search terms included a combination of keywords: ‘rocatinlimab’, ‘anti‐OX40’, ‘AMG 451’, ‘KHK4083’, ‘Atopic dermatitis’, ‘eczema’ and ‘randomised controlled trial’. Studies were restricted to those in the English language only. The details of the search strings are included in Supporting Information S2. Bibliographies of relevant articles were manually searched to identify further eligible studies.

Two independent reviewers (H.J.W. and L.S.W.) conducted a two‐stage screening process. First, titles and abstracts were screened to exclude irrelevant studies. Second, the full‐text articles were retrieved and assessed. Any disagreements were resolved through discussion and consulting the third reviewer (E.S.W).

### 2.2. Inclusion and Exclusion Criteria

Inclusion criteria include RCTs comparing rocatinlimab with placebo in adults with moderate‐to‐severe AD. Exclusion criteria were nonrandomised studies, studies evaluating other anti‐OX40 therapies, studies of nonatopic variants of dermatitis, animal studies and nonplacebo studies and non–English‐language publications.

### 2.3. Data Extraction

After importing all identified records into Covidence [15] and eliminating duplicates, two reviewers (H.J.W. and L.S.W) independently extracted the data using a standardised form. Extracted information included the authors’ names, study year, study design, location, population, number of participants, details of the intervention, study duration and outcomes of interest. Any disagreements were resolved through discussion and consultation with the third reviewer (E.S.W).

### 2.4. Risk of Bias

The methodological quality of each study included in this review was appraised using the Cochrane Risk of Bias (RoB 2) tool [16], assessing bias arising from five domains: randomisation, deviation from intended intervention, missing outcome data, outcome measurement and selection of reported results. Each study received an overall rating based on these assessments: ‘low risk’ if all domains were rated as low risk, ‘some concerns’ if at least one domain was rated as uncertain without being rated as high risk and ‘high risk’ if any single domain was rated as high risk. The Grading of Recommendations, Assessment, Development and Evaluation (GRADE) assessment [12] was conducted for each outcome to determine the certainty of evidence. Two reviewers (H.J.W. and L.S.W) independently assessed the risk of bias and certainty of evidence, with disagreements resolved through consensus or, where necessary, via discussion with a third reviewer (E.S.W).

### 2.5. Statistical Analysis

Pooled outcomes were measured using odds ratio (OR) with 95% confidence intervals (CI) with the Hartung–Knapp–Sidik–Jonkman (HKSJ) method for pooled outcomes involving three studies. The Wald‐type CI was used for pooled outcomes that include only two studies, as the HKSJ method may produce excessively wide intervals in this setting; however, Wald‐type intervals may be too narrow and were therefore interpreted cautiously [17]. Two‐sided *p* values of < 0.05 were considered statistically significant. Heterogeneity was assessed using I^2^ statistics, with *p* values < 0.10 and I^2^ values > 50% considered suggestive of substantial heterogeneity. All pooled outcomes were evaluated using the DerSimonian and Laird random‐effects model. Statistical analyses were conducted using Cochrane’s Review Manager 5.4 software [18]. Dose‐based subgroup analyses were conducted as exploratory clinical analyses for key efficacy and safety outcomes. They were not intended to identify definitive sources of heterogeneity or to establish dose–response relationships.

## 3. Results

### 3.1. Study Selection

**FIGURE 1:**
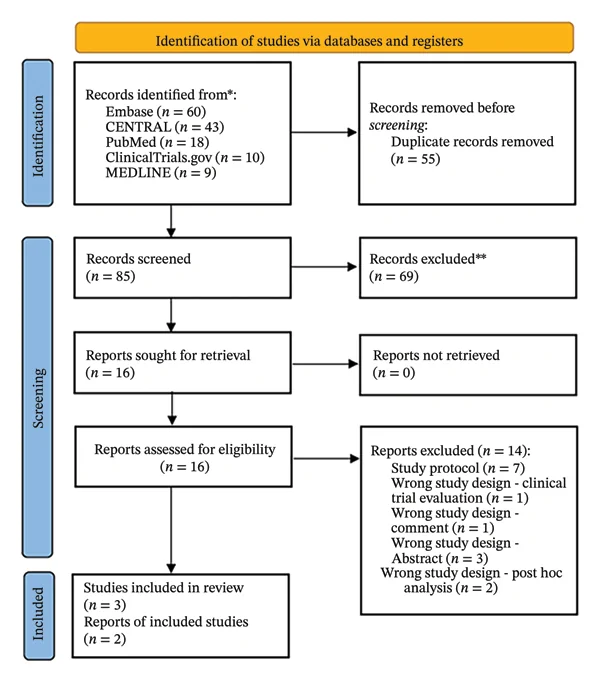
PRISMA diagram for the study selection process.

The initial database search yielded 140 records. After duplicate removal and title and abstract screening, 16 reports were retrieved for full‐text review. Of these, two reports describing three RCTs met the inclusion criteria and were included in this systematic review and meta‐analysis (Figure 1).

### 3.2. Study Characteristics

This systematic review included three RCTs that were conducted in 32 countries and 368 sites, involving 1767 adult patients with moderate‐to‐severe AD. These studies are one Phase 2b study and two Phase 3 trials (ROCKET‐IGNITE and ROCKET‐HORIZON) [8, 10], each using a multicenter, double‐blind, placebo‐controlled design. Participants were adults aged 18 years or older, with a mean age ranging from 37.6 to 38.5 years across studies. Across the Phase 2b and Phase 3 rocatinlimab studies, patients were required to have moderate‐to‐severe AD, defined by an EASI score ≥ 16, a vIGA‐AD score of 3 or 4 and ≥ 10% body surface area involvement. In the Phase 3 ROCKET studies, patients were additionally required to have a Worst Pruritus NRS score ≥ 4 [8, 10]. All three studies enrolled patients with prior inadequate response to topical therapies.

The included studies evaluated the efficacy and safety of rocatinlimab administered by subcutaneous injection. The dosage regimens varied across the trials. The Phase 2b study investigated rocatinlimab 150 and 600 mg every four weeks and 300 and 600 mg every two weeks [8]. The Phase 3 trials evaluated rocatinlimab at 150 and 300 every 4 weeks [11]. The studies had treatment durations ranging from 18 [8] weeks to 24 weeks [10], respectively, for the principal efficacy and safety outcomes. The study characteristics are summarised in Table 1.

**TABLE 1:** Study characteristics.

Study (year)	Study design	Location	Population	Number of participants	Mean disease duration	Baseline atopic dermatitis severity scores	Regimen of rocatinlimab	Duration of study
Guttman‐Yassky et al. (2023)	Multicentre, randomised, double‐blind, placebo‐controlled Phase 2b study	4 countries (65 sites)	Adults (aged ≥ 18 years) Female 58% Mean age: 38.0 years (SD 14.5) Moderate‐to‐severe atopic dermatitis	*N* = 274; Rocatinlimab (*n* = 217); Placebo, (*n* = 57)	16.2 years (SD 14.9)	vIGA‐AD score of 4 (44%) Mean EASI score 31.5 (SD 12.7) Mean SCORAD score 68.3 (SD 13.8) Mean affected body surface area: 56.7% (SD 23.4)	150 mg every 4 weeks; 600 mg every 4 weeks; 300 mg every 2 weeks; 600 mg every 2 weeks;	18 weeks

Guttman‐Yassky et al. (2026) ROCKET‐IGNITE	Multicentre, randomised, double‐blind, placebo‐controlled Phase 3 trials	19 countries (168 sites)	Adults (aged ≥ 18 years) Female 41% Mean age: 37.6 years (SD 14.6) Moderate‐to‐severe atopic dermatitis	*N* = 767; Rocatinlimab (*n* = 545); Placebo (*n* = 222)	22.1 years (SD 15.3)	vIGA‐AD score of 4 (35.9%) EASI score > 21 (70.5%) Mean affected body surface area: 44.0% (SD 21.2)	300 mg every 4 weeks; 150 mg every 4 weeks	24 weeks

Guttman‐Yassky et al. (2026) ROCKET‐HORIZON	Multicentre, randomised, double‐blind, placebo‐controlled Phase 3 trials	19 countries (151 sites)	Adults (aged ≥ 18 years) Female 45% Mean age: 38.4 years (SD 14.9) Moderate‐to‐severe atopic dermatitis	*N* = 726; Rocatinlimab (*n* = 543); Placebo, (*n* = 183)	24.8 years (SD 15.6)	vIGA‐AD score of 4 (38.3%) EASI score > 21 (70.2%) Mean affected body surface area: 44.1% (SD 22.2)	300 mg every 4 weeks	24 weeks

### 3.3. Treatment Efficacy

**FIGURE 2:**
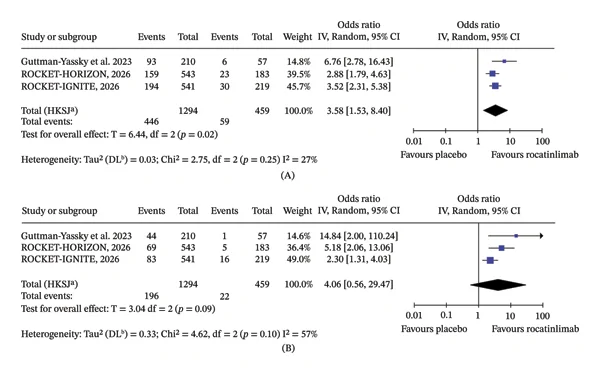
(A) EASI‐75 at Week 16. (B) vIGA‐AD of 0 or 1 at Week 16.

The pooled analysis of three RCTs indicated that subcutaneous rocatinlimab was associated with statistically significant achievement of at least 75% of baseline EASI (EASI‐75) at Week 16 compared with placebo (OR 3.58, 95% CI 1.53–8.40; *p* = 0.02; I^2^ = 27%; Figure 2A). However, rocatinlimab was not associated with achievement of a vIGA‐AD score of 0 or 1 at Week 16 compared with placebo (OR 4.06, 95% CI 0.56–29.47; *p* = 0.09; I^2^ = 57%; Figure 2B) [8, 10].

### 3.4. Safety Outcomes

**FIGURE 3:**
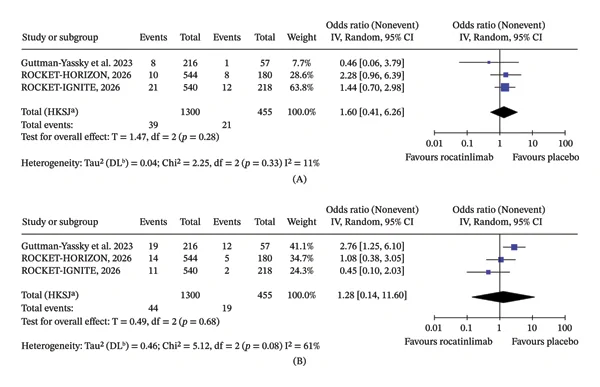
(A) Serious adverse events. (B) Treatment discontinuation due to adverse events.

Pooled ORs for serious adverse events (OR 1.60; 95% CI 0.41–6.26; *p* = 0.28; I^2^ = 11%; Figure 3A) and treatment discontinuation due to adverse events (OR 1.28; 95% CI 0.14–11.60; *p* = 0.68; I^2^ = 68%; Figure 3B) [8, 10] revealed no statistically significant differences between patients receiving rocatinlimab and placebo.

### 3.5. Subgroup Analysis

**FIGURE 4:**
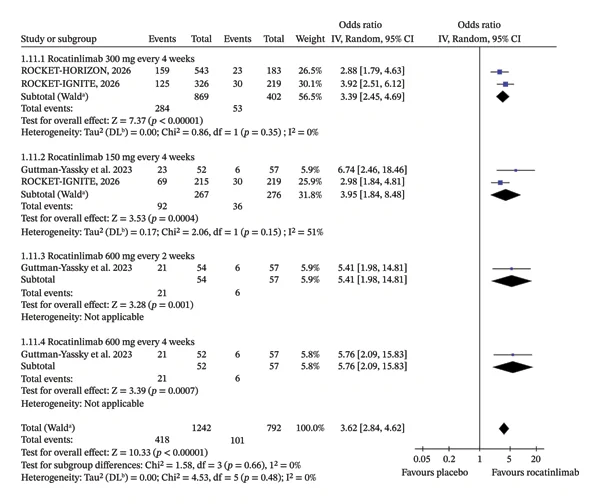
Subgroup analysis of EASI‐75 at Week 16 for different dosages of rocatinlimab.

**FIGURE 5:**
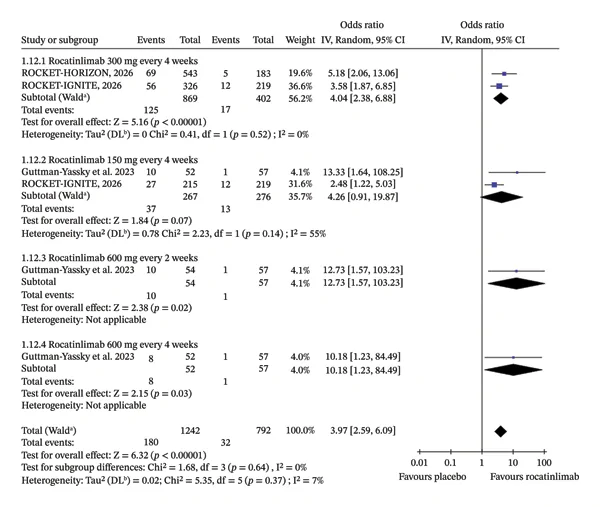
Subgroup analysis of vIGA‐AD of 0 or 1 at Week 16 for different dosages of rocatinlimab.

**FIGURE 6:**
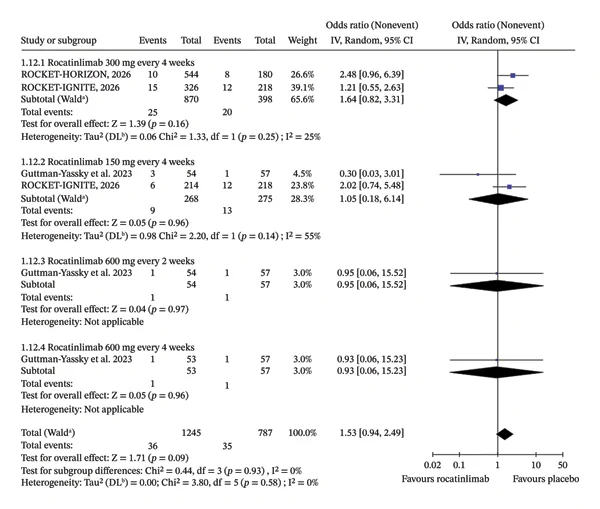
Subgroup analysis of serious adverse events for different dosages of rocatinlimab.

**FIGURE 7:**
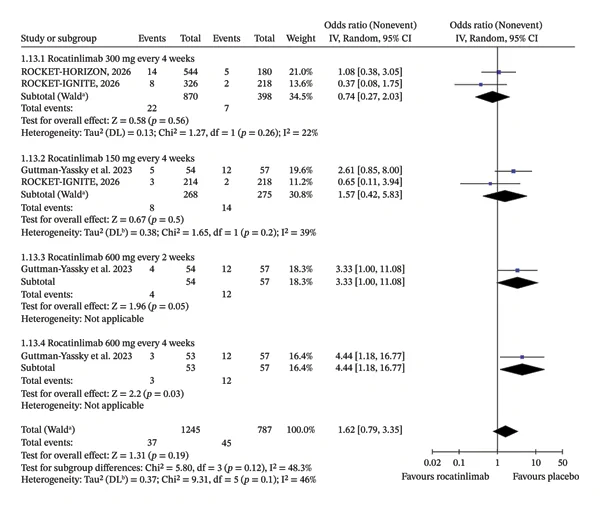
Subgroup analysis of treatment discontinuation due to adverse events for different dosages of rocatinlimab.

Exploratory dose‐based subgroup analyses were conducted for the key efficacy and safety outcomes to assess the directional consistency of treatment effects across the available dosing regimens. Among participants receiving different dose variations of rocatinlimab, there is a statistically significant improvement in the pooled OR for achieving EASI‐75 at Week 16 (Figure 4). For a vIGA‐AD score of 0 or 1 at Week 16, the pooled OR for all dosage variations except the dosage of rocatinlimab 150 mg every 4 weeks was statistically significant (Figure 5). For serious adverse events and treatment discontinuation due to adverse events, the pooled OR was not statistically different (Figures 6 and 7).

### 3.6. Risk of Bias

**FIGURE 8:**
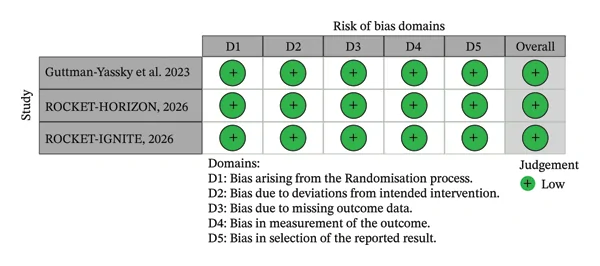
Summary of risk of bias for each included RCT.

All three RCTs were judged to have an overall low risk of bias across RoB 2 domains, indicating strong internal validity of the included trial evidence [12] (Figure 8.). Due to the limited number of included studies (< 10), funnel plot analysis for publication bias was not performed [11].

The GRADE assessment indicated moderate to high certainty for short‐term efficacy outcomes, but certainty for long‐term safety and durability of response was limited by short follow‐up duration and the small number of studies. (Supporting Information S3).

## 4. Discussion

This systematic review and meta‐analysis analysed randomised placebo‐controlled trials of subcutaneous rocatinlimab in adult patients with moderate‐to‐severe AD. Across three RCTs, including one Phase 2b study and two Phase 3 trials, rocatinlimab was associated with potentially improved short‐term efficacy outcomes with improved EASI‐75. These findings suggest potential short‐term improvement in clinical severity outcomes compared with placebo; however, the wide CI for vIGA‐AD 0/1 reflects substantial uncertainty in that estimate.

Several patient‐reported outcomes consistently reported across the included trials were not pooled in the meta‐analysis due to methodological heterogeneity across studies [17]. In all three trials, rocatinlimab was associated with improvements in the achievement of an EASI‐90 score, Worst Pruritus Numeric Rating Scale (WP‐NRS) and Dermatology Life Quality Index (DLQI) [8, 10]. However, the absolute proportions of patients achieving clinically meaningful thresholds are modest, with fewer than one‐third of rocatinlimab‐treated patients achieving a ≥ 4‐point WP‐NRS reduction and fewer than half achieving a clinically meaningful DLQI improvement across both trials [8, 10]. Differences in assessment timepoints and dosing regimens preclude pooling and limit the real‐world clinical significance of these findings. The available trial data demonstrate modest short‐term improvements in disease severity scores; however, they do not establish long‐term disease modification.

The proposed biological rationale for rocatinlimab is based on inhibition of the OX40 pathway, which is involved in T‐cell costimulation and inflammatory responses in AD [8, 10]. By targeting OX40‐expressing activated T cells, rocatinlimab may modulate immune pathways implicated in chronic skin inflammation [8, 10]. However, mechanistic plausibility may not be equivalent to clinically demonstrated disease modification. In particular, the prevention of recurrent inflammation via targeting OX40 as a long‐term solution for AD has yet to be supported by current clinical guidelines. Therefore, rocatinlimab’s proposed role in T‐cell modulation for the management of moderate‐to‐severe AD should be understood as a potential mechanistic rationale rather than evidence of long‐term clinical benefit [6, 9, 10].

In the pooled analysis of the included RCTs, there were no statistically significant differences between rocatinlimab and placebo in serious adverse events or treatment discontinuation due to adverse events. However, the short‐term study designs were not adequate to reliably detect rare, delayed, cumulative or long‐latency adverse outcomes. This limitation is particularly relevant in light of serious gastrointestinal adverse events, including ileal ulcers, and malignancies reported during the Phase 3 programme and subsequent safety‐related developments [8, 9, 14]. Although the absolute number of reported events was small and causality remains uncertain, these findings underscore that the available trial data cannot establish long‐term safety or a favourable overall benefit–risk profile.

The findings of this systematic review and meta‐analysis should therefore be interpreted in the context of the subsequent discontinuation of the rocatinlimab clinical development programme following safety review, for which Kyowa Kirin stated that the overall benefit–risk profile was no longer considered favourable in the studied populations [13, 14, 19]. Accordingly, the present results provide a synthesis of short‐term efficacy and trial‐reported safety outcomes rather than evidence supporting rocatinlimab as a clinically viable long‐term treatment option.

The current results are therefore best understood in a historical and mechanistic context. They provide a quantitative synthesis of short‐term efficacy and trial‐reported safety outcomes from available placebo‐controlled RCTs. The findings may remain informative for understanding the OX40 pathway in AD and for the future development of therapies targeting T‐cell costimulatory pathways. However, future work would require more robust long‐term safety surveillance and careful assessment of malignancy and immune‐related risks.

This study has a few limitations. First, only three RCTs were available, which limits the precision and robustness of the pooled estimates, restricts reliable exploration of between‐study variability and precludes meaningful assessment of publication bias. The small evidence base also limits the generalisability of the findings, as the included trials enrolled selected adults with moderate‐to‐severe AD under controlled trial conditions. Although HKSJ adjustment was applied to the primary pooled analyses to provide more conservative CIs and *p* values, Wald‐type CIs were used for subgroup‐specific pooled estimates involving only two studies [17]. Uncertainty remains because estimates of the overall effect and between‐study variation are inherently imprecise when few studies are available. Furthermore, although the GRADE assessment indicated moderate‐to‐high certainty for reported short‐term efficacy outcomes, this certainty applies only to the available trial evidence and does not establish durability of response, long‐term safety or applicability to broader clinical populations [17, 20, 21]. Second, the follow‐up duration of the included studies was insufficient to determine durability of response, recurrence after treatment discontinuation or long‐term safety. Third, differences in dosing regimens and study design may have contributed to variability across outcomes. The subgroup analyses reported in Section 3.5 were therefore conducted according to both dose and dosing frequency. However, these analyses should be interpreted cautiously because only one or two studies contributed to each regimen‐specific subgroup. For pooled subgroup estimates involving only two studies, Wald‐type CIs were used because the HKSJ method may produce excessively wide intervals in this setting. Nevertheless, Wald‐type CIs may be too narrow when few studies are included. Accordingly, the subgroup findings should be regarded as exploratory and hypothesis‐generating rather than confirmatory and should not be used to infer definitive differences between dosing regimens or establish a dose–response relationship. Fourth, the safety outcomes available for meta‐analysis were limited to those reported in the published RCTs and may not reflect subsequent programme‐level safety concerns. In addition, the review protocol was not prospectively registered in PROSPERO, which may introduce potential bias and limit methodological transparency. However, we minimised this risk by ensuring independent data screening, extraction and assessment by two reviewers [22]. Finally, the absence of head‐to‐head trials against approved systemic therapies limits conclusions regarding comparative efficacy and safety.

## 5. Conclusion

In conclusion, rocatinlimab demonstrated potential short‐term efficacy in improving EASI‐75 outcomes compared with placebo in adults with moderate‐to‐severe AD, although the more stringent efficacy endpoint of vIGA‐AD 0/1 was directionally favourable but did not reach statistical significance in this analysis. However, the available evidence does not establish a favourable long‐term safety profile, durable remission, prevention of recurrence or long‐term disease modification. Given the subsequent discontinuation of the clinical development programme following safety review, these findings should be interpreted cautiously and primarily as a synthesis of short‐term trial evidence rather than support for future clinical use.

## Author Contributions

Hong Jing Wang: conceptualisation, project administration, methodology design, data extraction, statistical analysis, writing–original draft and writing–review and editing. Leong Seng Wang: supervision and mentorship, project administration, methodology design, data extraction, statistical analysis, writing–original draft and writing–review and editing. Jordan Wei Lun Tan: methodology design, writing–original draft and writing–review and editing. Ee Syuen Wang: methodology design, statistical analysis, writing–original draft and writing–review and editing. Yan Ye Lee: writing–original draft and writing–review and editing.

## Funding

Open‐access publishing was facilitated by Monash University, as part of the Wiley–Monash University agreement via the Council of Australasian University Librarians.

## Ethics Statement

The authors have nothing to report.

## Conflicts of Interest

The authors declare no conflicts of interest.

## Supporting Information

Additional supporting information can be found online in the Supporting Information section.

## Supporting information


**Supporting Information 1** Supporting Information S1 PRISMA checklist.

**Supporting Information 2** Supporting Information S2 Search strategy.

**Supporting Information 3** Supporting Information S3 GRADE assessment.

## Data Availability

The data that support the findings of this study are available from the corresponding author upon reasonable request.
