# Supplementary material for: Efficacy and Safety of Subcutaneous Rocatinlimab in Adults With Moderate‐to‐Severe Atopic Dermatitis: A Systematic Review and Meta‐Analysis of Randomised Placebo‐Controlled Trials
Source: Dermatol Res Pract. 2026 Sep 20;2026:2160445. doi: 10.1155/drp/2160445 (PMC13591123; doi:10.1155/drp/2160445)
Supplement: Supplementary file 2 [file DRP-2026-2160445-s002.pdf]

Supplementary Material S2 Search strategy

Database	Query	Number of studies retrieved
Embase via Ovid	Keyword (Rocatinlimab or anti-OX40 or AMG 451 or AMG-451 or KHK4083 or "OX40 antibody") AND ("Atopic dermatitis" or eczema or "dermatitis, atopic") AND ("randomized controlled trial" or "randomised controlled trial" or "controlled clinical trial" or "placebo")<br>Limit to English language and "remove clinical trial (clinicaltrials.gov) records", year= Inception – January 2026<br>The search was performed in January 2026	60
Ovid Medline	Keyword (Rocatinlimab or anti-OX40 or AMG 451 or AMG-451 or KHK4083 or "OX40 antibody") AND ("Atopic dermatitis" or eczema or "dermatitis, atopic") AND ("randomized controlled trial" or "randomised controlled trial" or "controlled clinical trial" or "placebo")<br>Limit to English language, year= 1902 - January Week 4 2026<br>The search was performed in January 2026	9
Cochrane Central Register of Controlled Trials (CENTRAL)	Keyword (Rocatinlimab or anti-OX40 or AMG 451 or AMG-451 or KHK4083 or "OX40 antibody") AND ("Atopic dermatitis" or eczema or "dermatitis, atopic"). AND ("randomised controlled trial" or "controlled clinical trial" or "placebo").mp.<br>Limit to English language, year = 1890 – 2026<br>The search was performed in January 2026	43
Pubmed	Keyword (Rocatinlimab or anti-OX40 or AMG 451 or AMG-451 or KHK4083 or "OX40 antibody") AND ("Atopic dermatitis" or eczema or "dermatitis, atopic") AND ("randomized controlled trial" or "randomised controlled trial" or "controlled clinical trial" or "placebo")<br>Limit to English language, year= Inception – January 2026<br>The search was performed in January 2026	18
Clinical trial gov	Keyword (Rocatinlimab or anti-OX40 or AMG 451 or AMG-451 or KHK4083 or "OX40 antibody") AND ("Atopic dermatitis" or eczema or "dermatitis, atopic") AND ("randomized controlled trial" or "randomised controlled trial" or "controlled clinical trial" or "placebo")<br>Limit to English language, year= Inception – January 2026<br>The search was performed in January 2026	10
	Total	123
