# Supplementary material for: Efficacy and Safety of Subcutaneous Rocatinlimab in Adults With Moderate‐to‐Severe Atopic Dermatitis: A Systematic Review and Meta‐Analysis of Randomised Placebo‐Controlled Trials
Source: Dermatol Res Pract. 2026 Sep 20;2026:2160445. doi: 10.1155/drp/2160445 (PMC13591123; doi:10.1155/drp/2160445)
Supplement: Supplementary file 3 [file DRP-2026-2160445-s003.pdf]

Rocatinlimab compared to Placebo for Adults with Moderate-to-Severe Atopic Dermatitis

Patient or population: Adults with Moderate-to-Severe Atopic Dermatitis
Setting:
Intervention: Rocatinlimab
Comparison: Placebo

Outcomes	№ of participants (studies) Follow-up	Certainty of the evidence (GRADE)	Relative effect (95% CI)	Anticipated absolute effects	
				Risk with Placebo	Risk difference with Rocatinlimab
EASI-75 response at week 16	1753 (3 RCTs)	⨁⨁⨁⨁ High	**OR 3.58** (2.49 to 5.16)	129 per 1,000	**217 more per 1,000** (from 140 more to 304 more)
vIGA-AD score of 0 or 1 at week 16	1753 (3 RCTs)	⨁⨁⨁⨁ High	**OR 4.06** (1.69 to 9.75)	48 per 1,000	**122 more per 1,000** (from 30 more to 281 more)
Serious adverse events	1755 (3 RCTs)	⨁⨁⨁⨁ High	**OR 1.60** (0.41 to 6.26)	46 per 1,000	**26 more per 1,000** (from 27 fewer to 186 more)
Adverse event leading to treatment discontinuation	1755 (3 RCTs)	⨁⨁⨁⨁ High	**OR 1.28** (0.47 to 3.47)	42 per 1,000	**11 more per 1,000** (from 22 fewer to 90 more)

*__The risk in the intervention group__ (and its 95% confidence interval) is based on the assumed risk in the comparison group and the **relative effect** of the intervention (and its 95% CI).

CI: confidence interval; **OR:** odds ratio

GRADE Working Group grades of evidence
High certainty: we are very confident that the true effect lies close to that of the estimate of the effect.
Moderate certainty: we are moderately confident in the effect estimate: the true effect is likely to be close to the estimate of the effect, but there is a possibility that it is substantially different.
Low certainty: our confidence in the effect estimate is limited: the true effect may be substantially different from the estimate of the effect.
Very low certainty: we have very little confidence in the effect estimate: the true effect is likely to be substantially different from the estimate of effect.
